# Dietary intake in 6-year-old children from southern Poland: part 2 – vitamin and mineral intakes

**DOI:** 10.1186/s12887-014-0310-7

**Published:** 2014-12-24

**Authors:** Sylwia Merkiel, Wojciech Chalcarz

**Affiliations:** Food and Nutrition Department of the Eugeniusz Piasecki University School of Physical Education in Poznan, Poland Królowej Jadwigi 27/39 Street, 61-871 Poznan, Poland

**Keywords:** Children, Dietary intake, Vitamins, Minerals, Nutrition, Diet

## Abstract

**Background:**

Studies on vitamin and mineral intakes in children are very important: firstly because of the high prevalence of diet-related diseases and secondly because of the widespread consumption of highly processed foods which are characterised by high energy content and low density of essential nutrients. Therefore, the purpose of this study was to analyse vitamin and mineral intakes in 6-year-old children from southern Poland.

**Methods:**

Vitamin and mineral intakes were estimated from a three-day food record in 120 children, 64 girls and 56 boys, aged 6 years. Nutrient densities were estimated as amounts per 1000 kcal (4185 kJ) of energy intake. Statistical analysis was carried out by means of the IBM SPSS Statistics computer programme, version 19. The studied population was divided according to gender.

**Results:**

Intakes of folic acid (μg/1000 kcal) and vitamin C (mg, mg/1000 kcal) were significantly higher in girls. Nutrient densities for all vitamins were higher in girls, however, these results did not reach statistical significance. Intake of vitamin D was lower than EAR in all of the studied children. Intakes of sodium (mg) and zinc (mg) were significantly higher in boys. Intakes of the remaining minerals were higher in boys, however, these findings did not reach statistical significance. Nutrient densities for all minerals, except for sodium, zinc and manganese, were higher in girls. All of the studied children had sodium intakes above UL.

**Conclusions:**

Inadequate intakes of vitamin D, calcium and potassium in the studied 6-year-olds along with excessive sodium intake are the risk factors for developing osteoporosis and hypertension. To prevent these diseases in the studied children, educational programmes for both preschool staff and parents should be worked out and implemented.

## Background

In recent years, children’s diets in the developed countries, although high in energy, protein, fat and simple carbohydrates, are usually characterised by low vitamin and mineral content. Studies on school children showed that this is largely due to an increased consumption of fast foods, salty snacks, candy and soft drinks along with lower intakes of fruit, vegetables, grains and milk [1]. A study in 1.5-4.5-year-old children showed a decreasing intake of most micronutrients with increasing intake of non-milk extrinsic sugars [2]. It was reported to be especially disturbing in the case of iron and zinc which intakes fell below the Estimated Average Requirement in children who exceeded 24% of energy from non-milk extrinsic sugars [2]. Another study in German 2-18-year-olds showed a strong inverse association between vitamin and mineral intakes and intake of added sugars [3]. The authors [3] also reported that intakes of the following food groups: ‘meat, fish and eggs’, ‘fats and oils’, ‘grain’ and ‘fruits and vegetables’ fell with increasing intake of added sugars, with the strongest effect for ‘fruits and vegetables’. In Norwegian children and adolescents, intakes of almost all micronutrients and intakes of fruit and vegetables decreased with increasing content of added sugar [4]. Also in American preschoolers, intakes of micronutrients, as well as grains, vegetables, fruits, and dairy decreased with increasing added sugar intake [5].

The observed trends are highly unfavourable, since vitamins and minerals are essential nutrients for healthy growth and proper development of children. Adequate intakes of these micronutrients play also an important role in the prevention of diet-related diseases. Preventing these diseases should start as early as in childhood [6]. Since vitamins and minerals are biologically active, both their deficiency and excess have unfavourable influence on human health, especially in childhood when providing adequate vitamin and mineral intakes is crucial for the child’s physical, motor, mental and emotional development. It is particularly important for 6-year-old children who should attain school readiness [6] because in Poland and some European countries the age of six years is the last year of preschool attendance [7]. However, now in Poland, due to the changes in law, more and more children start school at the age of six years, similar to many European countries. Therefore, adequate intakes of vitamins and minerals are indispensable for the children to perform well at school. The studies showed that iodine, iron and folate are key nutrients for cognitive development [8,9] and that other vitamins and minerals such as vitamin B_12_ or zinc may also be important [8].

After the year 2000, no publications on vitamin and mineral intakes of only 6-year-old children were found. However, nine studies published in eleven articles [10-19] reported intakes of selected micronutrients in populations of wide age ranges including 6-year-olds. All of these studies varied in terms of the age range of the studied children and the number of vitamins and minerals which were analysed. In the Polish national study on a representative sample [19] and in the British population study called the National Diet and Nutrition Survey of young people aged 4–18 years [13], intakes of as many as eighteen micronutrients in a subgroup of 4-6-year-old children were reported. Another British population study called the National Diet and Nutrition Survey Rolling Programme 2008/2009 – 2011/2012 [10] reported the intakes of even more micronutrients, nineteen, but in a subgroup of a wider age range, that is from 4 to 10 years. Intakes of eleven micronutrients were presented for a representative sample of Belgian preschoolers including a subgroup of 4–6.5-year-old children [14]. Intakes of vitamins and minerals in groups of children of quite a narrow age ranges are available for Spanish 6-7-year-old children with intakes of nine micronutrients reported [15,16] and for Cretan children aged 5.7-7.6 years with intakes of thirteen micronutrients reported [18]. However, in the latter study, intakes of vitamins and minerals were expressed as nutrient density only. Another study on Spanish children reported intakes of sixteen micronutrients in a subgroup of 6-9-year-old children [17]. Even wider age range was applied in the National Health and Nutrition Examination Survey, 1999–2000, which reported intakes of eighteen micronutrients in the United States population, including a subgroup of 6-11-year-old children [11,12].

Taking into account the high prevalence of diet-related diseases and the widespread consumption of highly processed foods which are characterised by high energy content along with low density of essential nutrients, it is especially important to investigate vitamin and mineral intakes in children. Therefore, the purpose of this study was to analyse vitamin and mineral intakes in 6-year-old children from southern Poland, including nutrient density.

## Methods

### Subjects

The studied population comprised 120 children, 64 girls and 56 boys, who attended the last grade in the preschools associated with the Nowy Sącz League of Preschools and Schools Promoting Health in Nowy Sącz and the vicinity. The sampling design has been described in detail previously [7]. The study was approved by the Bioethics Committee of the Poznan University of Medical Sciences.

### Vitamin and mineral intakes

#### Data collection

To assess vitamin and mineral intakes in the studied children, parents and preschool staff were asked to keep a food record for three days: two preschool days and one free day (Sunday). Intakes of vitamin and mineral supplements were also taken into account. All the details about the method were described in the previous part of this article [7].

#### Dietary assessment

Vitamin and mineral intakes were calculated using the Dieta 4.0 computer programme which contains food composition database. The programme estimates the changes of nutritional value by calculating the losses of nutrients resulting from food processing. This programme has been described in details in the previous articles [7,20].

#### Comparison with dietary reference intakes

Vitamin and mineral intakes of the studied 6-year-olds were compared to dietary reference intakes for Polish population [21]. Intakes of vitamin A, B_1_, B_2_, B_6_, folic acid, vitamin B_12_, niacin, vitamin C, calcium, phosphorus, magnesium, iron, zinc, copper and iodine were compared to Estimated Average Requirement (EAR) [21]. Intakes of vitamin E, sodium and potassium were compared to Adequate Intake (AI) [21]. Dietary reference intakes for Polish population do not include EAR for vitamin D, thus intake of this vitamin was compared to EAR worked out by the Food and Nutrition Board of the Institute of Medicine [22]. Polish dietary reference intakes do not also include manganese, therefore intake of this mineral was compared to AI worked out by the Food and Nutrition Board of the Institute of Medicine [23].

Moreover, vitamin and mineral intakes of the studied children were compared to Tolerable Upper Intake Level (UL) when available. Sodium intake was compared to UL proposed by the National Food and Nutrition Institute in Warsaw [21]. Intakes of retinol, vitamin D, E, B_6_, folic acid, zinc, copper and iodine were compared to UL worked out by the Scientific Committee on Food [24] and intakes of niacin, vitamin C, calcium, phosphorus, iron and manganese – to UL worked out by the Food and Nutrition Board of the Institute of Medicine [22,23,25-27]. Magnesium intake was not compared to UL. This is because the UL was established for magnesium from nonfood sources and the studied children did not take magnesium supplements. Nutrient densities were estimated as amounts per 1000 kcal (4185 kJ) of energy intake.

### Statistical analysis

Statistical analysis was carried out by means of the IBM SPSS Statistics computer programme, version 19 (Chicago, IL, USA). The studied population was divided according to gender. Means, standard deviations (SD), medians and standard errors (SE) were calculated for vitamin and mineral intakes. The percentages of children with vitamin and mineral intakes below EAR were calculated to investigate the prevalence of inadequate intake. In addition, the percentages of children with vitamin and mineral intakes above UL were calculated to assess the risk of adverse health effects from excessive intake [28]. The percentages of children with nutrient intakes below AI were also presented, similarly to previous studies [14], however, it should be emphasised that AI cannot be used to estimate the prevalence of inadequate nutrient intakes for groups [28].

The same statistical methods as in the first part of the article were applied [7]. In the case of the qualitative variables, statistical significance was determined using Pearson’s chi-square test. For testing normality of the quantitative variables, the Shapiro-Wilk statistic was used. For normally distributed variables, the unpaired Student’s *t* test was applied to investigate statistically significant differences, whereas for skewed variables – the non-parametric Mann–Whitney *U* test was used. The level of significance in the case of all the tests was set at *P* ≤ 0.05.

## Results

Socio-demographic characteristics of the studied children and their families were presented in the previous article [7]. Table 1 shows vitamin intakes in the studied 6-year-old children and Table 2 presents the percentages of the studied 6-year-olds in the reference ranges for vitamin intakes. Intakes of folic acid (μg/1000 kcal) and vitamin C (mg, mg/1000 kcal) were significantly higher in girls. It is important to mention that nutrient densities for all vitamins were higher in girls, however, these results did not reach statistical significance. It is also noteworthy that intake of vitamin D was lower than EAR in all of the studied children.

**Table 1:** **Vitamin intakes in the studied 6-year-old children**

**Nutrient**	**Reference values**	**Girls (** ***n*** **= 64)**		**Boys (** ***n*** **= 56)**		**All children (** ***n*** **= 120)**		***P***	**Girls (** ***n*** **= 64)**		**Boys (** ***n*** **= 56)**		**All children (** ***n*** **= 120)**	
		**Mean**	**SD**	**Mean**	**SD**	**Mean**	**SD**		**Median**	**SE**	**Median**	**SE**	**Median**	**SE**
Vitamin A (retinol equivalent)														
(μg)	300^1^	1088	650	1062	550	1076	603	0.701	984	81	875	74	952	55
(μg/1000 kcal)	NA	619	402	562	298	592	357	0.232	538	50	457	40	500	33
Retinol														
(μg)	NA	505	566	489	233	498	441	0.303	411	71	433	31	430	40
(μg/1000 kcal)	NA	291	373	258	130	275	286	0.925	226	47	231	17	227	26
Beta-carotene														
(μg)	NA	3496	2454	3435	2769	3467	2595	0.504	2934	307	2428	370	2835	237
(μg/1000 kcal)	NA	1964	1323	1824	1486	1899	1397	0.269	1634	165	1380	199	1535	128
Vitamin D														
(μg)	10^1^	2.35	1.35	2.21	1.12	2.29	1.25	0.987	1.93	0.17	2.10	0.15	1.96	0.11
(μg/1000 kcal)	NA	1.33	0.75	1.17	0.62	1.25	0.70	0.333	1.12	0.09	1.06	0.08	1.08	0.06
Vitamin E														
(mg)	6^2^	7.23	2.59	7.10	2.31	7.17	2.45	0.788	6.97	0.32	6.94	0.31	6.95	0.22
(mg/1000 kcal)	NA	4.10	1.51	3.72	1.02	3.92	1.31	0.197	3.94	0.19	3.45	0.14	3.79	0.12
Vitamin B_1_														
(mg)	0.5^1^	0.999	0.331	1.016	0.371	1.007	0.348	0.879	0.948	0.041	0.954	0.050	0.949	0.032
(mg/1000 kcal)	NA	0.565	0.177	0.536	0.198	0.551	0.187	0.352	0.524	0.022	0.492	0.027	0.513	0.017
Vitamin B_2_														
(mg)	0.5^1^	1.693	0.468	1.668	0.492	1.681	0.478	0.636	1.654	0.059	1.672	0.066	1.659	0.044
(mg/1000 kcal)	NA	0.958	0.258	0.880	0.247	0.922	0.255	0.071	0.937	0.032	0.865	0.033	0.910	0.023
Vitamin B_6_														
(mg)	0.5^1^	1.51	0.36	1.50	0.37	1.51	0.36	0.986	1.49	0.04	1.45	0.05	1.48	0.03
(mg/1000 kcal)	NA	0.86	0.20	0.80	0.18	0.83	0.19	0.075	0.83	0.02	0.78	0.02	0.80	0.02
Folic acid														
(μg)	160^1^	201.3	43.6	202.9	39.1	202.0	41.4	0.925	196.9	5.4	198.1	5.2	197.5	3.8
(μg/1000 kcal)	NA	114.2	25.4	107.0	15.2	110.8	21.5	0.049	110.3	3.2	103.5	2.0	107.0	2.0
Vitamin B_12_														
(μg)	1.0^1^	3.52	2.21	3.59	1.25	3.55	1.82	0.318	3.24	0.28	3.44	0.17	3.29	0.17
(μg/1000 kcal)	NA	2.02	1.45	1.89	0.65	1.96	1.14	0.713	1.77	0.18	1.66	0.09	1.76	0.10
Niacin														
(mg)	6^1^	12.30	3.31	12.24	3.15	12.27	3.23	0.875	11.42	0.41	11.43	0.42	11.43	0.29
(mg/1000 kcal)	NA	7.01	1.93	6.47	1.56	6.76	1.78	0.094	6.85	0.24	6.14	0.21	6.36	0.16
Vitamin C														
(mg)	40^1^	77.9	36.6	66.9	31.0	72.8	34.4	0.051	73.0	4.6	59.8	4.1	65.5	3.1
(mg/1000 kcal)	NA	43.9	20.3	35.3	16.3	39.9	18.9	0.003	40.7	2.5	32.6	2.2	35.7	1.7

NA – not available; *P* – significance.

^1^EAR; ^2^AI.

**Table 2:** **The percentages of the studied 6-year-old children in the reference ranges for vitamin intakes**

**Nutrient**	**Girls (** ***n*** **= 64)**	**Boys (** ***n*** **= 56)**	**All children (** ***n*** **= 120)**	***P***
	**%**	**%**	**%**	
Vitamin A (retinol equivalent)				
Below EAR	0.0	0.0	0.0	#
Retinol				
Above UL	1.6	1.8	1.7	0.924
Vitamin D				
Below EAR	100.0	100.0	100.0	#
Vitamin E				
Below AI	34.4	41.1	37.5	0.450
Above UL	0.0	0.0	0.0	#
Vitamin B_1_				
Below EAR	1.6	0.0	0.8	0.350
Vitamin B_2_				
Below EAR	0.0	0.0	0.0	#
Vitamin B_6_				
Below EAR	0.0	0.0	0.0	#
Above UL	0.0	0.0	0.0	#
Folic acid				
Below EAR	12.5	8.9	10.8	0.530
Above UL	1.6	1.8	1.7	0.924
Vitamin B_12_				
Below EAR	0.0	0.0	0.0	#
Niacin				
Below EAR	1.6	1.8	1.7	0.924
Above UL	26.6	14.3	20.8	0.099
Vitamin C				
Below EAR	10.9	16.1	13.3	0.409
Above UL	0.0	0.0	0.0	#

*P* – significance;

# – *P* cannot be calculated when percentage is 0.0 or 100.0.

Table 3 presents mineral intakes in the studied 6-year-old children and Table 4 shows the percentages of the studied 6-year-olds in the reference ranges for mineral intakes. Intakes of sodium (mg) and zinc (mg) were significantly higher in boys. Also, intakes of the remaining minerals were higher in boys, however, these findings did not reach statistical significance. Although statistically insignificant, it is important to mention that nutrient densities for all minerals, except for sodium, zinc and manganese, were higher in girls. Moreover, all of the studied children had sodium intakes above UL and substantial percentage of them had intake of manganese above UL. Substantial percentages of the studied 6-year-olds had intake of calcium below EAR and intake of potassium below AI.

**Table 3:** **Mineral intakes in the studied 6-year-old children**

**Nutrient**	**Reference values**	**Girls (** ***n*** **= 64)**		**Boys (** ***n*** **= 56)**		**All children (** ***n*** **= 120)**		***P***	**Girls (** ***n*** **= 64)**		**Boys (** ***n*** **= 56)**		**All children (** ***n*** **= 120)**	
		**Mean**	**SD**	**Mean**	**SD**	**Mean**	**SD**		**Median**	**SE**	**Median**	**SE**	**Median**	**SE**
Calcium														
(mg)	800^1^	673	201	712	227	691	213	0.327	640	25	663	30	652	19
(mg/1000 kcal)	NA	378	96	374	99	376	97	0.799	371	12	374	13	371	9
Phosphorus														
(mg)	410^1^	992	199	1050	227	1019	213	0.201	971	25	1021	30	994	19
(mg/1000 kcal)	NA	560	84	553	86	557	84	0.866	553	10	548	11	551	8
Magnesium														
(mg)	110^1^	218	42	228	47	223	45	0.468	217	5	217	6	217	4
(mg/1000 kcal)	NA	123	18	120	19	122	18	0.386	124	2	123	3	123	2
Sodium														
(mg)	1000^2^	2876	557	3194	650	3024	620	0.023	2960	70	3125	87	2989	57
(mg/1000 kcal)	NA	1634	299	1685	265	1658	284	0.326	1630	37	1656	35	1645	26
Potassium														
(mg)	3100^2^	2450	471	2511	498	2478	482	0.493	2491	59	2453	66	2468	44
(mg/1000 kcal)	NA	1383	199	1326	212	1356	206	0.128	1377	25	1342	28	1366	19
Iron														
(mg)	4^1^	9.0	2.0	9.4	2.3	9.2	2.1	0.315	8.8	0.2	9.0	0.3	8.9	0.2
(mg/1000 kcal)	NA	5.1	1.0	5.0	1.0	5.0	1.0	0.446	4.8	0.1	4.7	0.1	4.8	0.1
Zinc														
(mg)	4^1^	6.7	1.3	7.2	1.3	6.9	1.3	0.041	6.6	0.2	7.1	0.2	6.8	0.1
(mg/1000 kcal)	NA	3.8	0.5	3.8	0.5	3.8	0.5	0.290	3.7	0.1	3.9	0.1	3.8	0.0
Copper														
(mg)	0.3^1^	0.88	0.18	0.93	0.19	0.90	0.19	0.134	0.86	0.02	0.91	0.03	0.87	0.02
(mg/1000 kcal)	NA	0.50	0.08	0.49	0.08	0.49	0.08	0.704	0.49	0.01	0.48	0.01	0.49	0.01
Manganese														
(mg)	1.5^2^	3.00	0.79	3.23	0.77	3.11	0.79	0.106	2.90	0.10	3.17	0.10	3.09	0.07
(mg/1000 kcal)	NA	1.70	0.41	1.71	0.38	1.70	0.40	0.829	1.69	0.05	1.62	0.05	1.66	0.04
Iodine														
(μg)	65^1^	129.3	31.9	138.4	35.7	133.5	33.9	0.144	130.5	4.0	136.0	4.8	132.3	3.1
(μg/1000 kcal)	NA	73.8	19.6	73.4	18.4	73.6	19.0	0.925	71.5	2.4	72.2	2.5	71.7	1.7

NA – not available; *P* – significance.

^1^EAR; ^2^AI.

**Table 4:** **The percentages of the studied 6-year-old children in the reference ranges for mineral intakes**

**Nutrient**	**Girls (** ***n*** **= 64)**	**Boys (** ***n*** **= 56)**	**All children (** ***n*** **= 120)**	***P***
	**%**	**%**	**%**	
Calcium				
Below EAR	76.6	66.1	71.7	0.203
Above UL	0.0	0.0	0.0	#
Phosphorus				
Below EAR	0.0	0.0	0.0	#
Above UL	0.0	0.0	0.0	#
Magnesium				
Below EAR	0.0	0.0	0.0	#
Sodium				
Below AI	0.0	0.0	0.0	#
Above UL	100.0	100.0	100.0	#
Potassium				
Below AI	92.2	83.9	88.3	0.160
Iron				
Below EAR	0.0	0.0	0.0	#
Above UL	0.0	0.0	0.0	#
Zinc				
Below EAR	1.6	0.0	0.8	0.350
Above UL	3.1	5.4	4.2	0.543
Copper				
Below EAR	0.0	0.0	0.0	#
Above UL	0.0	0.0	0.0	#
Manganese				
Below AI	0.0	0.0	0.0	#
Above UL	46.9	57.1	51.7	0.261
Iodine				
Below EAR	0.0	0.0	0.0	#
Above UL	0.0	0.0	0.0	#

*P* – significance;

# – *P* cannot be calculated when percentage is 0.0 or 100.0.

## Discussion

### Methodological remarks

To assess vitamin and mineral intakes in the studied 6-year-olds, we chose an estimated food record. This method has the advantage of eliminating the problem of forgetting and improves estimation of portion size because the information is recorded at consumption [29]. Since food record carries a higher respondent burden [29], we chose a three-day period. Moreover, this method was most frequently used in the previous studies on vitamin and mineral intakes in children [14,18,30]. The methods used in other studies included a four-day estimated food record [10], a seven-day weighed food record [13], food frequency questionnaire [15,16], one 24-hour dietary recall [11,12,19] or a combination of 24-hour dietary recall and food frequency questionnaire [17]. The differences in vitamin and mineral intakes observed in the studied 6-year-olds and in the previously studied children [10-19] are surely caused by methodological differences. However, most probably various food habits explain most of these differences. For example, vitamin D intake was very low in all of the previously studied children [10,14,15,17,30] irrespective of the method of dietary assessment and intake of vitamin A was much higher in Polish children, both in the studied 6-year-olds and in the previously studied 4-6-year-olds [19], in comparison to children from other countries, which may be explained by Polish food habits.

### Vitamin intakes

Mean intakes of all the analysed vitamins, except for vitamin D, were well above the reference values which implies low risk of inadequate intakes in the studied 6-year-old children. It is particularly favourable in the case of vitamins B_1_, B_2_, B_6_, B_12_ and folic acid. This is because inadequate intakes of these vitamins are linked to elevated plasma homocysteine concentration which, in turn, is related to increased risk of cardiovascular diseases, such as coronary heart disease and stroke [31,32]. This is of great importance to the studied children because of their excessive intake of saturated fatty acids and animal protein along with inadequate intake of polyunsaturated fatty acids, which pose the risk of developing cardiovascular diseases, as reported in the previous article [7]. Therefore, inadequate intakes of B vitamins would aggravate the risk of developing these diseases in the studied 6-year-olds. Additionally, the same effect would have inadequate intakes of antioxidant vitamins and so it is highly favourable that intakes of β-carotene and vitamins E and C pose low risk of inadequate intake in the studied children.

The only major concern in the studied 6-year-olds is low intake of vitamin D which implies high risk of inadequate intake. Similar or even lower intakes of vitamin D were observed in 4-10-year-old [10] and 7-year-old [30] British children, and in Spanish 6-9-year-old children [17]. Also, Belgian 4–6.5-year-olds [14] were characterised by lower intake of vitamin D compared to the studied 6-year-olds and almost all of them had intakes lower than Belgian recommendations. Such low intake of vitamin D is highly disconcerting because it may adversely affect the studied children’s bones and teeth [33,34]. Moreover, studies imply that deficiency of this vitamin has negative impact on insulin resistance and β cell function [35-37] increasing the risk of diabetes. Although vitamin D is synthesised as a result of exposure to solar ultraviolet-B irradiation [38], in Polish climatic conditions such low intake of vitamin D is unlikely to be compensated by cutaneous synthesis of this vitamin. It is highly surprising that the staff of preschools promoting health failed to spread the recommendation of eating fish frequently which would prevent inadequate vitamin D intakes in the studied children.

In the previous studies on vitamin intakes, 6-year-old children were included in groups of children of various age ranges [10-19], therefore, direct comparison to other studies is not possible. Only nutrient density may be compared directly because it is not dependent on total energy intake. In Cretan 5.7-7.6-year-old children [18] nutrient densities were higher than in the studied 6-year-olds for six out of nine analysed vitamins, that is for vitamin B_1_, B_2_, B_6_, folic acid, niacin and vitamin C [18]. Serra-Majem et al. [17] and Glynn et al. [30] also analysed nutrient densities but in the former study the amounts of vitamins were given per 1000 kJ of energy intake while in the latter study only statistically significant differences according to gender were analysed and no values were presented.

Although vitamin intakes cannot be compared directly to other studies, it is interesting to note that intake of vitamin A in the studied 6-year-old children was much higher than in British 4-6-year-olds [13], but also much higher than in older groups of children: 4-10-year-old [10] and 7-year-old [30] British children, 6-7-year-old [15,16] and 6-9-year-old [17] Spanish children, and even in the United States 6-11-year-olds [12]. In Polish 4-6-year-olds [19], intake of vitamin A was also lower in comparison to the studied 6-year-old children, but it was higher than in children from other countries irrespective of age. In comparison to those studies in which intakes of retinol [10,13,19,30] and β-carotene [13,19,30] were analysed, intakes of these nutrients were also the highest in the studied children as well as in the previously studied Polish 4-6-year-olds [19]. This high intake of vitamin A in the studied 6-year-old children may be explained by high consumption of carrot juice which is very popular in Poland, especially among children and adolescents. There are many brands of carrot juice in Poland addressed particularly to the youngest consumers. Moreover, the studied children attended preschools promoting health where the staff tried to implement the habit of eating vegetables. The region where the studied children lived is not affluent and carrot which is not an expensive vegetable was often consumed by the children at preschool. The other habit which surely increased vitamin A intake is daily use of butter which is in Poland the most popular fat to spread on sandwiches.

In the previous studies, which reported vitamin intakes in children of similar age to the studied 6-year-olds, the populations of children were divided according to gender [10,12-14,17-19,30], except for the Spanish 6-7-year-olds [15,16] whose intakes were analysed according to the city where the children lived. However, statistically significant differences according to gender were tested only in two of those studies [14,30].

It was expected to find many statistically significant differences in vitamin intakes between the studied girls and boys. However, it turned out not to be true in the studied children. Glynn et al. [30] found statistically significant differences in intakes of five vitamins in English 7-year-olds, while Huybrechts and De Henauw [14] found significant differences in intakes of four vitamins in Belgian 4–6.5-year-olds.

In the previous studies, vitamin intakes were usually reported to be higher in boys than in girls [10,12-14,17]. Only in 7-year-old English children [30], girls were characterised by higher intake of vitamin A, retinol and β-carotene, and in 4-6-year-old Polish children [19] girls were characterised by higher intakes of vitamin A, β-carotene, vitamin B_2_, niacin and vitamin C. However, these differences were minor and statistically insignificant. Quite opposite, Huybrechts and De Henauw [14] found statistically significantly lower intakes of vitamins D, B_1_, B_2_ and C in Belgian 4–6.5-year-old girls in comparison to their male peers, while Glynn et al. [30] in the group of English 7-year-olds found statistically significantly lower intakes of vitamins B_1_, B_2_, B_6_, niacin and folic acid in females in comparison to males.

Among the previous studies on vitamin intakes in children of similar age to the studied 6-year-olds, nutrient densities for vitamins were analysed only in three of them [17,18,30]. Unlike in the studied 6-year-olds, nutrient densities for vitamins were not always higher in girls. Glynn et al. [30] found higher nutrient densities in English 7-year-old girls only for vitamin A, retinol and β-carotene, however, all these differences were statistically significant. Smpokos et al. [18] reported higher nutrient densities in Cretan 5.7-7.6-year-old girls for as many as six out of nine analysed vitamins but Serra-Majem et al. [17] reported higher nutrient densities in 6-9-year-old Spanish girls for only three out of ten vitamins. However, neither Smpokos et al. [18] nor Serra-Majem et al. [17] tested statistically significant differences according to gender.

### Mineral intakes

It is surprising that the studied 6-year-old children are at risk of inadequate calcium intake. The importance of drinking milk to children’s bone health has been spread throughout the Polish society for many years and even television has broadcast a series of spots promoting the habit of daily milk drinking. Also, the producers of dairy products use this recommendation in the commercials. Moreover, the children attended preschools promoting health and therefore it would seem obvious that basic nutritional guidelines should be promoted by the preschool staff. Most of the preschool staff and the studied children’s parents knew that high intake of milk and dairy products in childhood prevents osteoporosis [39,40] and that milk and dairy products are rich sources of calcium [41,42]. The adverse effect of inadequate calcium intake may be aggravated by the abovementioned inadequate vitamin D intake and by quite high phosphorus intake. Although the studied children did not exceed the UL, phosphorus intake was much higher than calcium intake. This may disturb the proportion of calcium to phosphorus which should be about 1.2 : 1 in children’s diet according to the Polish recommendations [43].

Another adverse characteristic of the studied 6-year-olds’ diets was excessive intake of sodium found in all of the studied children. Exceeding sodium UL poses the risk of developing hypertension, particularly when taking into account quite low potassium intake. Although EAR for potassium has not been established, mean intake below AI shows the need to increase intake of this mineral to prevent hypertension in the studied 6-year-olds. It is unexpected that the preschool staff failed to convince the parents of the necessity to reduce salt intake and did not implement this simple rule during the preparation of preschool meals. It is even more surprising when taking into account that the majority of both the preschool staff and the studied children’s parents knew that high salt intake increases the risk of hypertension [39,40]. These findings confirm the necessity to implement programme aimed at reducing salt intake as proposed in the previous article [44].

It is also disconcerting that manganese intake exceeded UL in about a half of the studied children. However, bioavailability of this mineral from food sources have been found to be affected by other dietary factors [23], such as phytate which reduces the efficiency of absorption of manganese [45]. Therefore, blood manganese concentration should be measured in the studied children to conclude whether manganese intake is excessive.

Similarly to vitamin intakes, also mineral intakes cannot be compared directly to the results of other studies because of the age differences among the studied populations of children. However, it is important to note that sodium intake in the studied children was higher than in 4-6-year-old Polish children [19] and much higher than in 4-6-year-old British children [13] and 4–6.5-year-old Belgian children [14]. Moreover, it was higher even than in older children: British 7-year-olds [30], as well as 6-7-year-old [15,16] and 6-9-year-old [17] Spanish children. Nutrient densities for calcium, potassium and iron in the studied children were lower than nutrient densities for these minerals in Cretan 5.7-7.6-year-olds [18]. However, nutrient density for sodium in the studied 6-year-olds was much higher than in Cretan 5.7-7.6-year-olds [18]. Nutrient densities for other minerals were not analysed by Smpokos et al. [18].

Contrary to expectations and similarly to vitamin intakes, there were statistically significant gender differences in the intakes of only two minerals in the studied 6-year-olds. Among those studies in which differences in mineral intakes were tested according to gender, only two of them reported statistically significant differences [14,30].

Higher intakes of all the analysed minerals in the studied 6-year-old boys compared to their female peers, were also observed in all of the previously studied children [10,11,13,14,17,19,30]. Only calcium intake in Polish 4-6-year-old boys [19] and magnesium intake in 4–6.5-year-old Belgian boys [14] were not higher than in their female peers. Moreover, Huybrechts and De Henauw [14] and Glynn et al. [30] reported these differences to be statistically significant, except for selenium intake in 7-year-old English boys [30].

Higher nutrient densities for most of the analysed minerals in the studied 6-year-old girls compared to their male peers were not as noticeable as in the case of vitamins. Smpokos et al. [18] reported higher nutrient densities for three out of four minerals in Cretan 5.7-7.6-year-old girls but Serra-Majem et al. [17] – only for two out of six minerals in Spanish 6-9-year-old girls.

## Conclusions

In conclusion, inadequate intakes of vitamin D, calcium and potassium in the studied 6-year-olds along with excessive sodium intake are the risk factors for developing osteoporosis and hypertension. To prevent these diseases in the studied children, educational programmes for both preschool staff and parents should be worked out and implemented.
